# The Thirty-Fifth Annual Session of the Baltimore College Dental Surgery

**Published:** 1874-11

**Authors:** 


### EDITORIAL. ETC.

---

*The Thirty-fifth Annual Session of the Baltimore College of Dental Surgery.*—The preliminary course of lectures of this institution began October 15th, the introductory having been delivered by Professor E. Lloyd Howard.

The friends of the College will be gratified to learn that the present session bids fair to be one of the most successful in its history, the number of students present at the preliminary course being more than one half larger than for several years past.

Germany, England, South America, Guatemala, Jamaica, Canada, and the West Indies, together with nearly all the Southern, and a number of the Northern States are already represented; and as the majority of students enter during the first week of the regular course, which begins November 2nd, there is every reason for believing that the present session will prove a most prosperous one for this, the oldest Dental College in the world.

Among the students is another lady from Germany, who, there is every reason to suppose, will do as much honor to herself and the institution, as the two former female graduates; both of these, Misses Foeking & Jacobi, having graduated with high honors, are now very successfully practicing their profession in Berlin, Prussia.

Although some have endeavored to make the admission of females into the profession a matter for censure, and, in order to advance the interests of others less worthy, have gone so far as to reflect upon the Baltimore College yet the Faculty, so far from regretting their action in this respect, are very well satisfied to affix their names to the diplomas of all of the opposite sex, who may prove as well qualified at the final examination, as those who have already graduated from the Baltimore College of Dental Surgery. None have so far applied for matriculation, who are not ladies in every sense of the word, besides being well educated and bringing the highest testimonials.

Since the last session the College Building has been enlarged nearly one half, and presents as fine an appearance as any institution of the kind in the country. In addition to the new part of the building, both the Lecture Room and Infirmary have been enlarged. The new addition contains the Museum, a Dissecting room, and en Operating room for the extraction of teeth and the administration of anæsthetic agents, altogether separate from the Infirmary proper.

The Infirmary practice is very large and steadily increasing, the College Building being situated in the best location in the city for such a practice. New appliances have also been added, and every care taken to insure a thorough course of instruction.

